# Epidemiological characteristics of breakthrough varicella cases among students in Jiulongpo District, Chongqing: implications for vaccination strategies

**DOI:** 10.3389/fimmu.2025.1594598

**Published:** 2025-06-18

**Authors:** Huixian Zhou, Qianjin Long, Hechuan Xie, Yuan Yao, Chunyan Deng

**Affiliations:** Department of Infectious Diseases Prevention and Control, Center for Disease Control and Prevention of Jiulongpo District, Chongqing, China

**Keywords:** varicella, breakthrough cases, epidemiological characteristics, students, vaccination

## Abstract

**Objective:**

This study aimed to investigate the epidemiological characteristics and determinants of breakthrough varicella (BV) cases in student populations, identify key factors influencing breakthrough infection intervals, and provide recommendations for vaccination strategy optimization.

**Methods:**

A retrospective analysis was conducted on varicella cases and vaccination records among students in Jiulongpo District, Chongqing (2019-2023). Descriptive epidemiology methods were employed to characterize BV cases, with linear regression modeling assessing correlations between post-vaccination breakthrough intervals, primary immunization age, and the interval between two doses.

**Results:**

Among 970 BV cases (27.25% of total cases), significant difference was observed: (1) Temporal distribution exhibited bimodal epidemic peaks (May-June and October-December), with seasonal variation in breakthrough proportions (spring 28.35%, summer 25.33%, autumn 29.67%, winter 23.97%); (2) Geographic analysis revealed differential distribution across urban (28.07%), urban-rural fringe (23.04%), and rural areas (28.97%); (3) Population stratification showed highest proportions in kindergarten children (37.45%), followed by primary school (33.63%), middle school (24.75%), and high school students (6.87%). The linear regression analysis demonstrated that single-dose BV cases showed a negative correlation between post-vaccination breakthrough interval and primary immunization age (r=-0.384, P<0.05); two-dose BV cases exhibited dual negative correlations with both primary immunization age (r=-0.225, P<0.05) and the interval between two doses (r=-0.228, P<0.05).

**Conclusion:**

The rising incidence of varicella breakthrough infections necessitates transitioning to a two-dose regimen. To reduce the risk of breakthrough infections in student populations, we recommend timely administration of the first varicella vaccine dose at 12 months of age, followed by a second booster dose as early as possible.

## 1 Introduction

Varicella, caused by the varicella-zoster virus (VZV), is an acute contagious respiratory disease characterized by generalized pruritic vesicles that typically resolve within one week, with severe complications occurring in a minority of cases (1, 2). Primary VZV infection predominantly affects young children. Following initial infection, the virus establishes lifelong latent infection in peripheral neurons and may reactivate as herpes zoster during immunosuppression, creating a distinctive “infection-latency-reactivation” epidemiological cycle (3, 4). Varicella is the third most reported vaccine-preventable infectious disease in China, following tuberculosis and influenza, posing significant socioeconomic burdens on families and public health systems (5). School-aged children constitute the primary affected population, representing 82.88% of total reported cases in mainland China from 2016 to 2019 (6). Outbreaks in kindergartens and schools frequently persist for months with substantial disruptions, necessitating stringent control measures such as the 14-day home isolation for infected students and 21-day medical surveillance for exposed classes mandated by Chongqing’s Municipal Education Commission and Health Commission. In China, clinical diagnosis is prioritized over time-consuming laboratory confirmation for varicella case identification to facilitate early detection, containment, and isolation (7). As defined in the *Diagnosis and Treatment Plan for Varicella (2023 Edition) (* 8) published by the National Health Commission of the People’s Republic of China, clinical criteria include: (1) pruritic cutaneous eruptions progressing sequentially from erythematous macules and papules to vesicles and crusts, typically originating on the trunk and head before spreading systemically, with or without accompanying fever and headache; (2) atypical presentations coupled with epidemiological exposure history (contact with varicella/herpes zoster cases within 3 weeks prior to symptom onset).

Varicella vaccine (VarV) remains the most effective and cost-efficient strategy for preventing and controlling varicella outbreaks (9), significantly reducing disease incidence in school populations during epidemics (10). The World Health Organization (WHO) recommends routine childhood immunization in countries where varicella poses substantial public health burdens (11). But if varicella vaccination coverage stays at a medium level (30% – 70%) for a long time, varicella’s incidence and mortality rates will rise due to infection age delay (12). Although China’s domestically developed VarV has been widely used since its introduction in 2000 (13), it has not yet been incorporated into the national immunization program (14). A 2020 nationwide survey revealed suboptimal vaccination rates: 52.72% of children aged 1–14 years received one dose, while only 11.43% completed the two-dose regimen (15). Vaccine effectiveness against VZV was 81% for single-dose and 92% for two-dose recipients (16), with corresponding breakthrough varicella (BV) incidence rates of 8.5 (95% CI: 5.3–13.7) and 2.2 (95% CI: 0.5–9.3) cases per 1000 person-years (17). Persistent BV occurrences are attributed to multifactorial challenges including suboptimal dosing schedules, cold chain inconsistencies, and host immunological variability (18–22).

BV is a wild-type VZV infection occurring > 42 days post-vaccination, primarily attributable to primary vaccine failure or antibody waning (23). BV typically presents with milder symptoms than natural infection, featuring fewer lesions and shorter duration (24–26). However, delayed recognition and isolation of BV cases during early disease stages pose substantial challenges to outbreak containment (27). In recent varicella outbreaks in Jiulongpo District, BV cases have shown a high-frequency occurrence, though their exact proportion among total varicella cases remains unclear. This study aims to conduct an in-depth analysis of the epidemiological characteristics of BV cases, comprehensively investigating their temporal, spatial, and demographic distributions to provide foundational data for epidemic control. By comparing breakthrough cases with non-breakthrough cases in terms of epidemiological features, we seek to reveal the potential impact of vaccination on disease transmission dynamics. Additionally, despite limitations in existing data, this study will employ statistical methods to explore potential associations between breakthrough infection intervals and primary immunization age or vaccination intervals, analyzing how immunization schedules influence the timing of breakthrough infections. Ultimately, these findings will provide a scientific basis for optimizing vaccination strategies, safeguarding student health, and identifying critical pathways to enhance the effectiveness of varicella prevention and control measures.

## 2 Materials and methods

### 2.1 Study area

**Figure 1:**
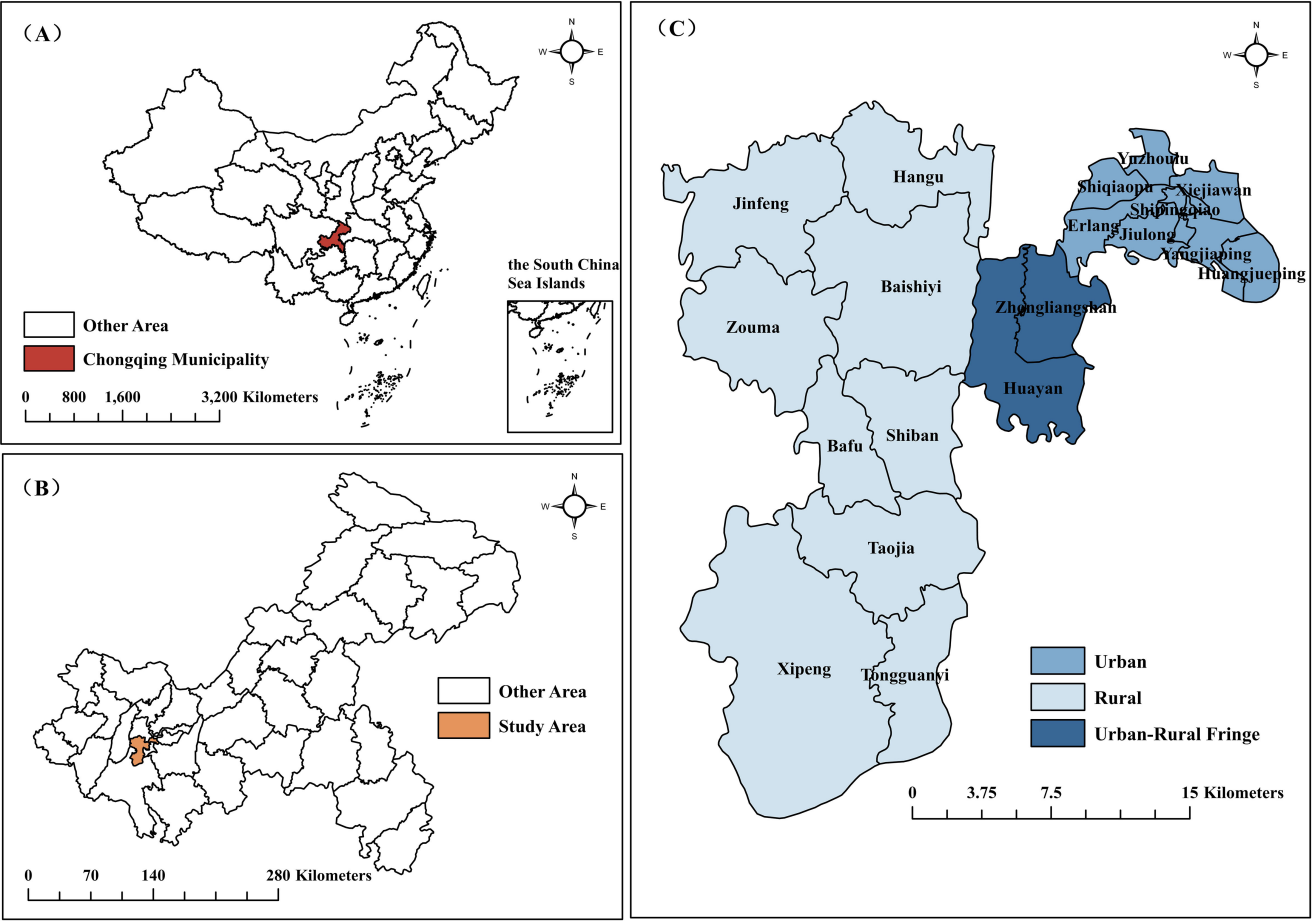
Geographical location of Jiulongpo District. **(A)** The People’s Republic of China (population: 1.408 billion); **(B)** Chongqing Municipality (Population: 31.90 million); **(C)** Jiulongpo District (Population: 1.54 million).

Jiulongpo District, strategically positioned as a transitional zone between Chongqing’s urban core and western mountainous peripheries, was selected for its epidemiological representativeness in southwestern China. Spanning 432 km² with 19 towns, it sustains a permanent population of 1.54 million and achieved a 2024 regional GDP of ¥206 billion alongside an average annual disposable income of ¥54 409, ranking second economically in Chongqing (28). The district’s healthcare infrastructure comprises 934 hierarchical medical institutions, with the Health Commission implementing a “15-minute basic healthcare service radius” through optimally distributed community health stations (≤1.2 km service coverage), ensuring broad healthcare accessibility. Geographically, the area encapsulates three socioecological strata: urban areas (8 towns, 62.62% population) characterized by high-density residential clusters with advanced medical resources; the urban-rural fringe areas (2 towns, 20.80% population) serving as dynamic hubs for population mobility and economic transition; and rural areas (9 towns, 16.59% population) marked by geographic dispersion and healthcare access disparities. This tripartite structure mirrors China’s urbanization challenges, offering a natural experiment to examine breakthrough varicella transmission dynamics across differential population mobility patterns, as shown in **Figure 1**.

### 2.2 Data resources

The varicella case data in this study were sourced from the China Information System for Disease Control and Prevention (CISDCP), a nationwide internet-based infectious disease surveillance platform established by the Chinese government in 2004. Although varicella is not included in China’s statutory infectious disease management framework, Chongqing has classified it as a Category C infectious disease since 2014, mandating healthcare facilities to report all suspected, clinically diagnosed, or laboratory-confirmed varicella cases to CISDCP within 24 hours. Reported case information includes ID number, name, gender, age, occupation (with specific school and class details for students), address, date of onset, date of diagnosis, and case classification (suspected, clinical, or laboratory-confirmed cases). Vaccination histories were retrieved from the “Chongqing Epidemic Prevention and Control Information System”, developed by the Chongqing Center for Disease Control and Prevention, which allows tracing of individual immunization records using ID numbers as the search criterion. The student population denominator was derived from the *Statistical Bulletin on National Economic and Social Development of Jiulongpo Distric*t, *Chongqing*, published by the Jiulongpo District Bureau of Statistics.

### 2.3 Define

- *Breakthrough varicella (BV)* (23): Varicella infection occurring > 42 days after VarV administration.
- *Primary immunization age:* Age at first VarV dose.
- *Interval between two doses:* Time between two VarV doses.
- *Breakthrough interval*: The time interval between varicella vaccination and the occurrence of varicella infection. Calculation formula: Breakthrough interval (years) = Date of varicella onset - Date of last varicella vaccination.

### 2.4 Inclusion and exclusion criteria

**Figure 2:**
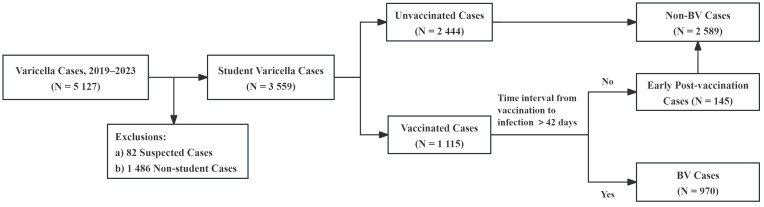
BV cases filtering process.

Inclusion criteria: (1) the address was Jiulongpo District, Chongqing; (2)the diagnosis was varicella; (3) the onset time was between January 2019 and December 2023; (4) the case classification was clinical cases or laboratory-confirmed cases; (5) population category was kindergarten children or students (excluding university students). Exclusion criteria: (1) the case classification was a suspected case; (2) non-student populations. The filtering process is shown in **Figure 2**.

### 2.5 Analyses

Data were collated using Excel 2021 (Microsoft). Descriptive epidemiology characterized spatiotemporal distributions of BV cases. Seasonal patterns were analyzed via seasonal index (SI) and coefficient of variation (CV):


$$SI=\frac{Mean\;number\;of\;BV\;cases\;in\;the\;same\;month\;of\;each\;year}{Mean\;number\;of\;BV\;cases\;in\;all\;months}\;(\text{SI}>1\text{ indicates high}-\text{incidence month})$$

$$CV=\frac{Standard\;deviation}{Mean}\;(\text{Lower CV reflects stronger seasonality})$$

R 4.4.1 software performed statistical analyses. Categorical variables were expressed as counts (%) and continuous variables as mean ± standard deviation (SD) or median (interquartile range). For group comparisons, categorical variables were analyzed using chi-square (χ²) test, while non-normally distributed continuous variables were assessed with Mann-Whitney U test (two-group comparisons) or Kruskal-Wallis H test (multi-group comparisons). Linear regression assessed: (1) Association between primary immunization age and breakthrough interval in single-dose BV;(2) Relationships among primary immunization age, interval between two doses, and breakthrough interval in two-dose BV; Statistical significance was set at *P < 0.05.*

## 3 Result

### 3.1 Epidemiological characteristics

From 2019 to 2023, a total of 3 559 varicella cases were reported among students in Jiulongpo District, Chongqing, with an average annual incidence rate of 308.97 per 100 000 population. Middle school students exhibited the highest annual incidence (395.60/100 000), while kindergarten children had the lowest (188.02/100 000). Temporally, the overall incidence demonstrated a fluctuating decline, decreasing from 512.70/100 000 in 2019 to 195.25/100 000 in 2023 (61.90% reduction), as shown in **Table 1**. BV cases accounted for 27.25% (970/3 559) of total cases, with their proportion showing an upward trend from 20.12% in 2019 to 37.68% in 2023 (87.28% increase). Specifically, single-dose BV cases constituted 82.99% (805/970) of all BV cases, compared to 17.01% (179/970) for two-dose cases, as shown in **Table 2**.

**Table 1:** Varicella incidence per 100 000 student population.

Year	Incidence (1/100 000)				
	Kindergarten	Primary school	Middle school	High school	Total
2019	328.91	714.85	452.41	389.33	512.70
2020	162.45	247.85	364.64	343.76	262.01
2021	220.85	386.16	493.28	342.25	356.56
2022	116.07	194.30	383.52	244.39	218.35
2023	111.81	159.52	284.14	285.29	195.25
Mean	188.02	340.53	395.60	321.00	308.97

**Table 2:** Proportion of breakthrough varicella cases among total varicella cases.

Year	Non-BV cases	BV cases [n(%)]						Total
		Single-dose		Two-dose		Total		
2019	901	211	(92.95)	16	(7.05)	227	(20.12)	1128
2020	459	120	(88.24)	16	(11.76)	136	(22.86)	595
2021	599	197	(84.19)	37	(15.81)	234	(28.09)	833
2022	334	154	(79.38)	40	(20.62)	194	(36.74)	528
2023	296	123	(68.72)	56	(31.28)	179	(37.68)	475
Total	2 589	805	(82.99)	165	(17.01)	970	(27.25)	3 559

BV, breakthrough varicella.

#### 3.1.1 Time distribution

**Figure 3:**
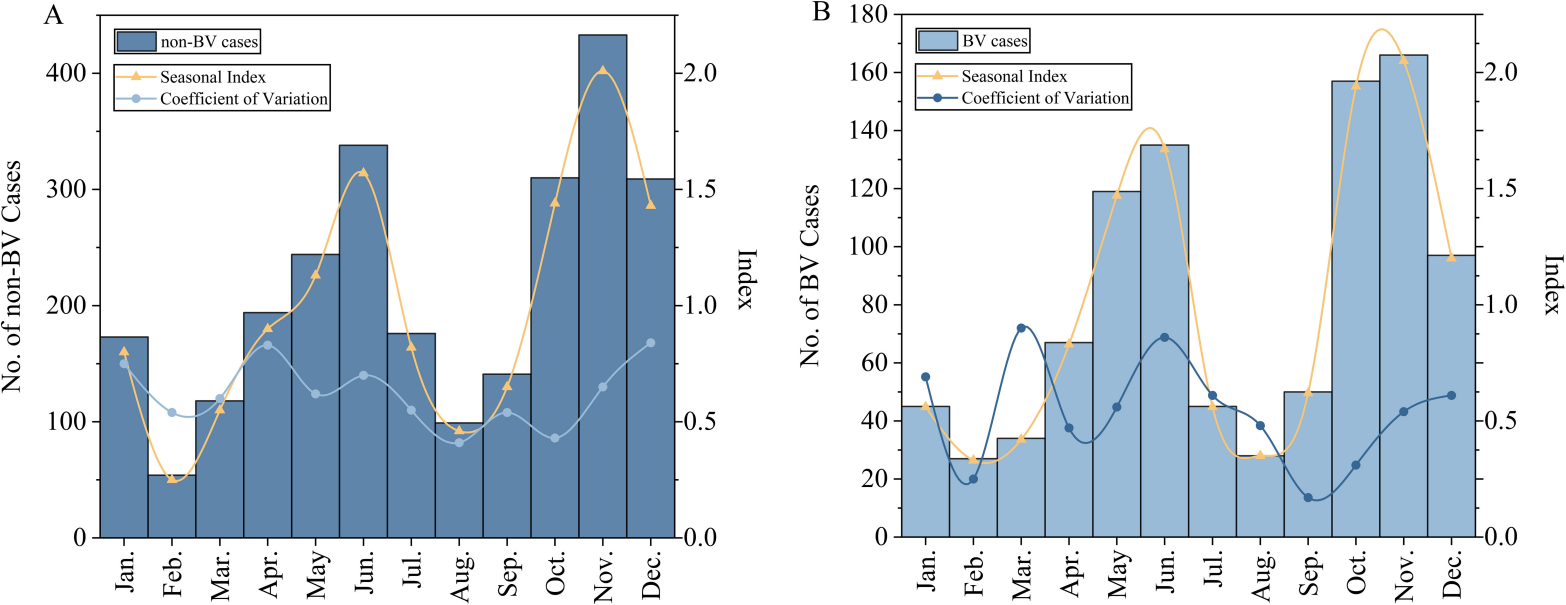
Number of varicella cases and seasonal characteristics. **(A)** non-BV cases; **(B)** BV cases.

From 2019 to 2023, both non-BV and BV cases among the student exhibited the same incidence peaks in May–June and October–December. During these two periods, the SI were consistently greater than 1. Notably, October showed a smaller CV, indicating more pronounced seasonality, as shown in **Figure 3**. Seasonal analysis revealed 220 (28.35%), 208 (25.33%), 373 (29.67%), and 169 (23.97%) BV cases occurring in Spring (March–May), Summer (June–August), Autumn (September–November), Winter (December–February), respectively, with significant seasonal variation (*χ²=9.539, P<0.05*), as shown in **Table 3**.

**Table 3:** Univariate analysis of breakthrough varicella cases among students in Jiulongpo District.

Variable	Non-BV cases [n(%)]		BV cases [n(%)]		*χ^2^*	*P*
Gender					0.015	0.902
Male	1 422	(72.66)	535	(27.34)		
Female	1 167	(72.85)	435	(27.15)		
Population classification					199.818	0.001
Kindergarten	334	(62.55)	200	(37.45)		
Primary school	1 034	(66.37)	524	(33.63)		
Middle school	611	(75.25)	201	(24.75)		
High school	610	(93.13)	45	(6.87)		
Region					8.802	0.012
Urban	1 317	(71.93)	514	(28.07)		
Urban-rural fringe	578	(76.96)	173	(23.04)		
Rural	694	(71.03)	283	(28.97)		
Season					9.539	0.023
Spring (March–May)	556	(71.65)	220	(28.35)		
Summer (June–August)	613	(74.67)	208	(25.33)		
Autumn (September–November)	884	(70.33)	373	(29.67)		
Winter (December–February)	536	(76.03)	169	(23.97)		

BV, breakthrough varicella.

#### 3.1.2 Regional distribution

**Figure 4:**
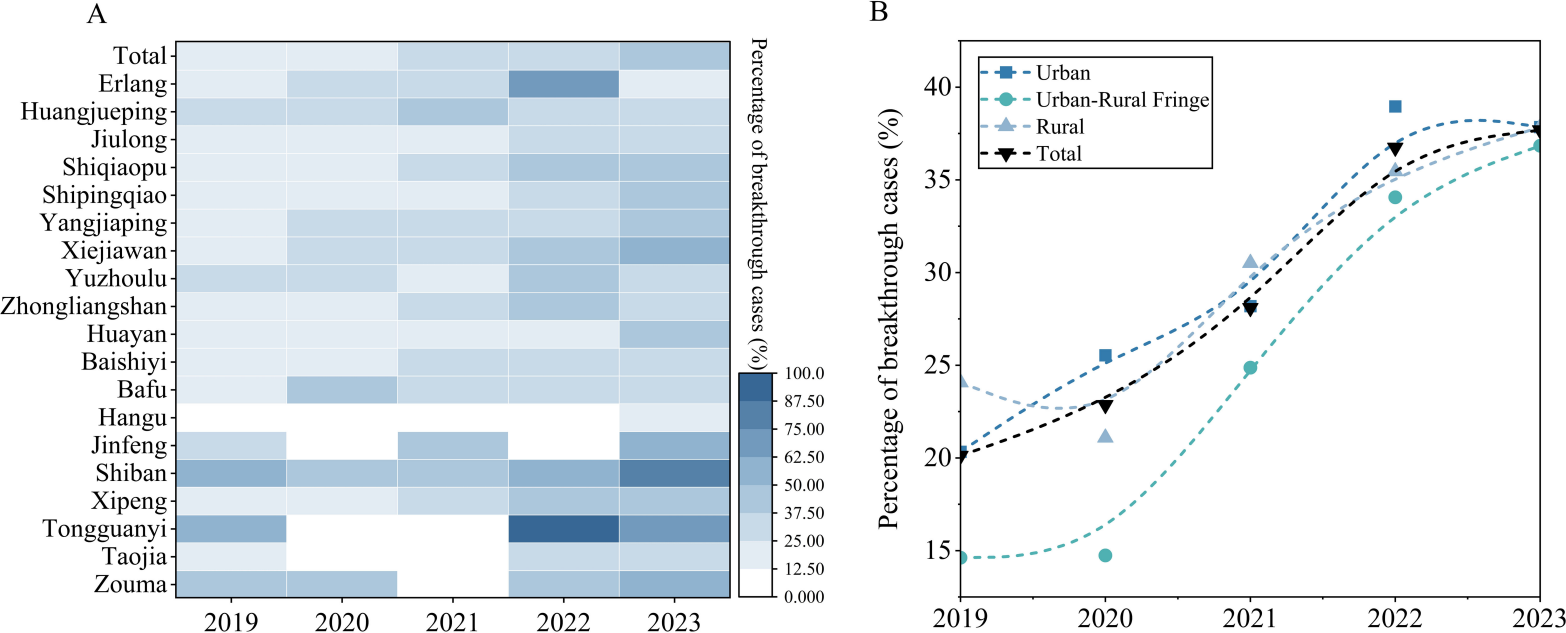
Regional distribution of BV cases. **(A)** Towns; **(B)** Regions.

Urban areas, urban-rural fringe, and rural areas reported 1 831, 751, and 977 varicella cases, respectively. BV cases were reported across all 19 towns. The top five towns by case count accounted for 49.79% of total BV cases: Huayan (106), Xipeng (101), Shiqiaopu (96), Yangjiaping (94), and Xiejiawan (86). The highest BV proportions occurred in Tongguanyi (53.49%), Shiban (48.39%), Zouma (42.25%), Jinfeng (35.80%), and Huangjueping (33.33%), as shown in **Figure 4A**. The proportions of BV cases in urban, urban-rural fringe, and rural areas demonstrated an upward trend, as shown in **Figure 4B**. Urban, urban-rural fringe, and rural areas accounted for 514 (28.07%), 173 (23.04%), and 283 (28.97%) BV cases, respectively, showing significant spatial variation (*χ^2^ = 8.802, P<0.05*), as shown **Table 3**.

#### 3.1.3 Demographic distribution

The BV cases comprised 535 males and 435 females (gender ratio: 1.23:1), with no significant gender-based difference in case proportions (*χ^2^ = 0.015, P=0.902*). Significant variation emerged across population classification: kindergarten children (37.45%), primary school students (33.63%), middle school students (24.75%), and high school students (6.87%) exhibited differential BV proportions (*χ^2^ = 199.818, P<0.001*), as shown in **Table 3**.

### 3.2 Breakthrough interval analysis

**Figure 5:**
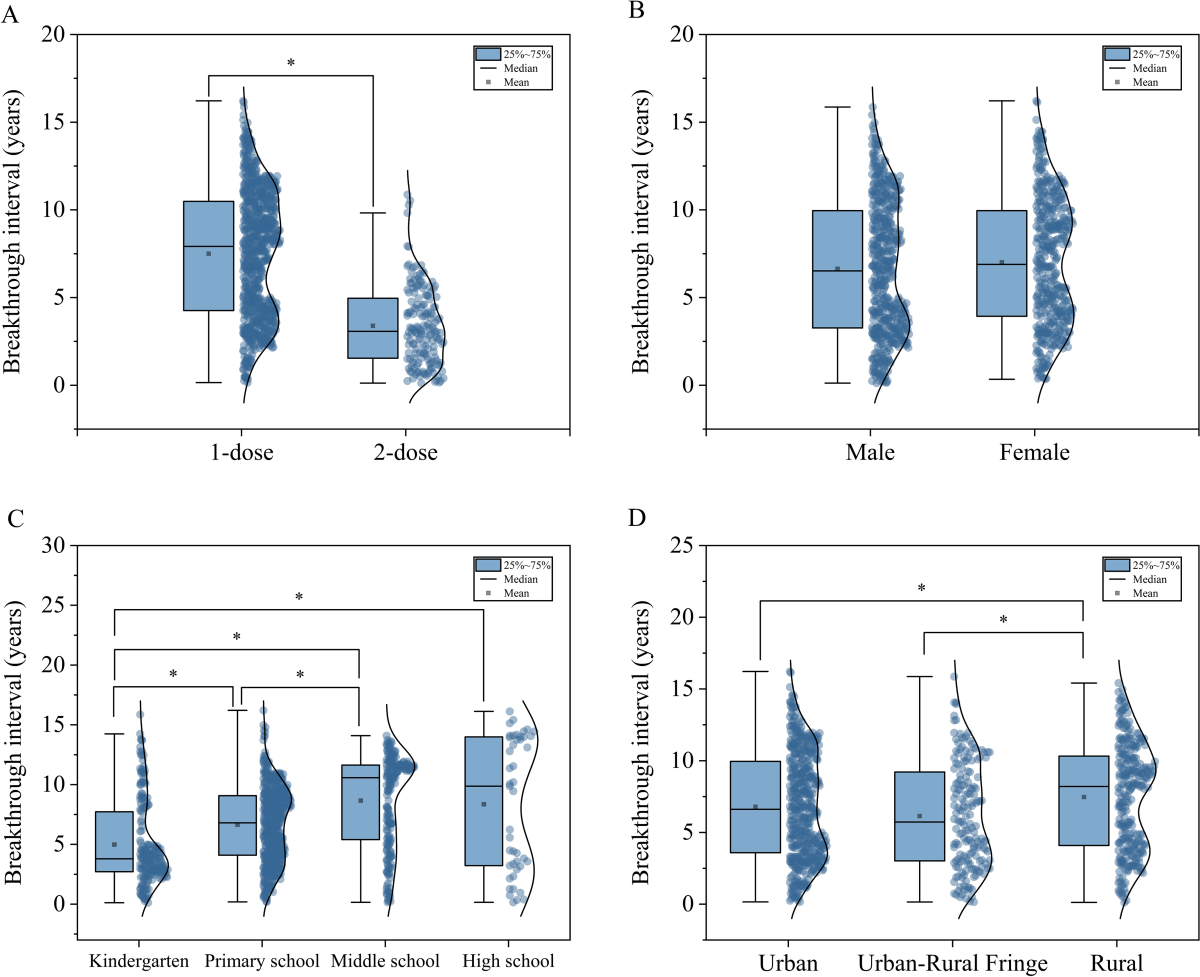
Distribution of breakthrough intervals in BV cases by category. **(A)** Dose; **(B)** Gender; **(C)** Population classification; **(D)** Geographic region. * means P<0.05.

The breakthrough intervals of 970 cases ranged from 45 days to 16.22 years, with a median of 6.73 (3.58–9.95)years. Among these, single-dose cases (n=805) had a median interval of 7.92 (4.25–10.49) years, significantly longer than the 3.07 (1.52–5.00) years observed in two-dose cases (n=165) (*Z = 12.949, P < 0.05*), as shown in **Figure 5A**. Males and females showed comparable median intervals of 6.52 (3.27–9.95) years and 6.89 (3.93–9.95) years, respectively, with no statistically significant difference (*Z = -1.603, P > 0.05*), as shown in **Figure 5B**. Kindergarten children, primary school students, middle school students, and high school students exhibited median breakthrough intervals of 3.79 (2.71–7.75), 6.81 (4.09–9.08), 10.58 (5.34–11.64), and 9.87 (3.19–14.00) years, respectively, with statistically significant differences across population groups (*H = 108.812, P < 0.05*), as shown in **Figure 5C**. Significant regional variations were observed in median breakthrough intervals: 6.61 (3.56–9.96) years in urban areas, 5.73 (3.02–9.23) years in the urban-rural fringe, and 8.21 (4.09–10.32) years in rural areas (*H = 14.195, P < 0.05*), as shown in **Figure 5D**.

**Figure 6:**
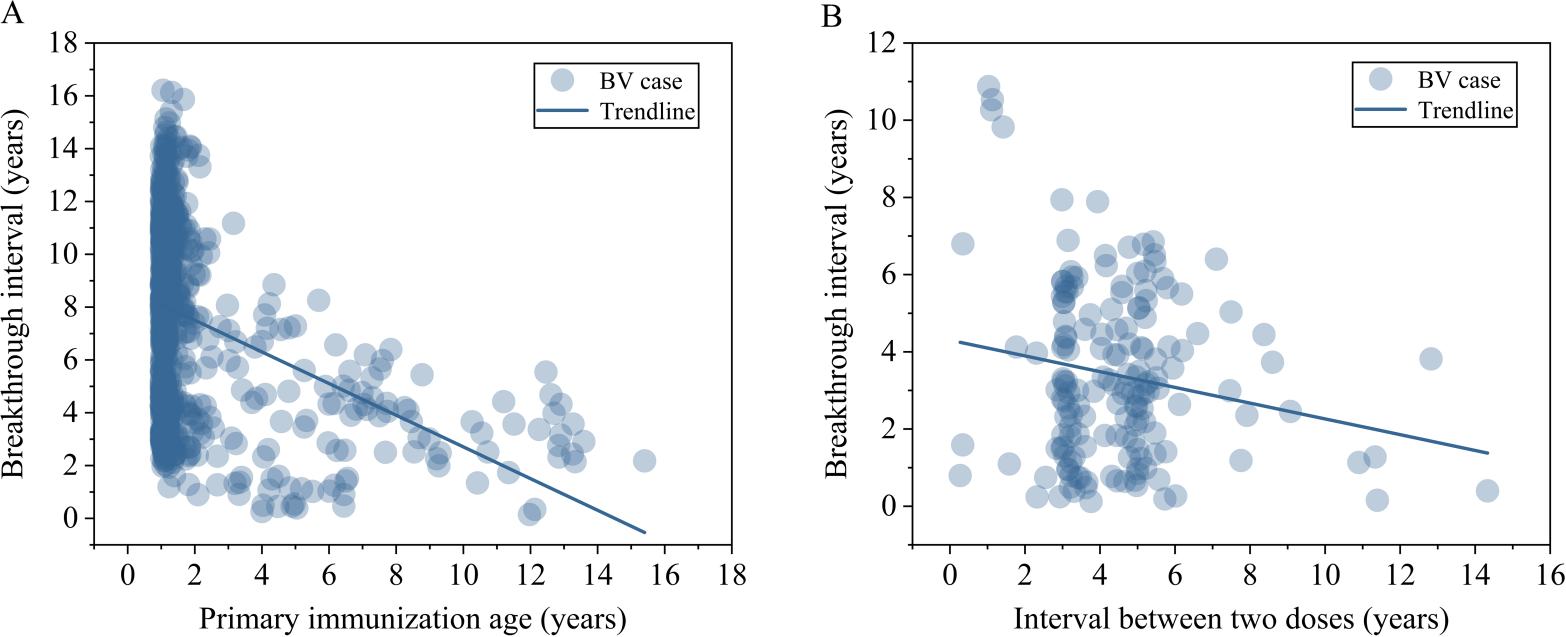
Relationships between breakthrough intervals, primary immunization age, and vaccination intervals in student BV cases. **(A)** Single-dose: Association of breakthrough intervals with primary immunization age; **(B)** Two-dose: Association of breakthrough intervals with vaccination intervals.

Among single-dose cases, the age at primary immunization ranged from 1 to 15.40 years, with a mean of 2.01 ± 2.31 years and a median of 1.15 (1.05–1.48) years. Breakthrough intervals showed a significant negative correlation with primary immunization age (*r= -0.384, t = -11.785, P < 0.05*), as shown in **Figure 6A** and **Table 4**. Primary immunization age for two-dose BV cases ranged from 1 to 12.67 years, with a mean of 1.42 ± 1.36 years and a median of 1.12 (1.04–1.25) years. The interval between the two doses ranged from 99 days to 14.33 years, with a mean of 4.46 ± 1.97 years and a median of 4.37 (3.14–5.19) years. Breakthrough intervals correlated negatively with both primary immunization age (*r = −0.225, t = −2.938*, *P* < 0.05) and the interval between two doses (*r = −0.228, t = −2.981, P < 0.05*), as shown in **Figure 6B** and **Table 4**.

**Table 4:** Linear regression analysis of breakthrough intervals among students in Jiulongpo District.

Variables	Nonnormalized coefficient		*r*	*t*	*P*	95% confidence interval	
	*B*	*S.E.*				Lower	Upper
Single-dose breakthrough infection (n=805)							
constant	3 177.595	56.696		56.047	0.000	3 066.306	3 288.884
Primary immunization age	-218.955	18.578	-0.384	-11.785	0.000	-255.423	-182.487
Two-dose breakthrough infection (n=165)							
constant	1 838.771	173.329		10.609	0.000	1 496.495	2 181.047
Primary immunization age	-135.654	46.17	-0.225	-2.938	0.004	-226.826	-44.483
Interval between two doses	-0.25	0.084	-0.228	-2.981	0.003	-0.416	-0.084

B, non-standardized regression coefficient; S.E., standard error; r, standardization coefficient.

## 4 Discussion

From 2009 to 2023, varicella incidence among students in Jiulongpo District demonstrated a declining trend, potentially associated with improved vaccination coverage, strengthened school-based prevention measures, and heightened public health awareness. However, the rapid increase in the proportion of BV cases highlights emerging challenges in current immunization strategies.

The bimodal peaks of BV cases in May-June and October-December among Jiulongpo students align with Chongqing’s varicella epidemic pattern (29). These periods coincide with school terms and significant climatic fluctuations, potentially contributing to reduced immunity and heightened transmission risks. The lower BV proportions during summer and winter vacations underscore school terms as critical windows for outbreak control (30). Geographically, the proportion of BV cases in the urban-rural fringe was significantly lower than in urban and rural areas. While regional vaccination coverage data remain unavailable to directly elucidate the drivers of these disparities, rural areas exhibited notably longer median breakthrough intervals (8.21 years) compared to urban (6.61 years) and urban-rural fringe areas (5.73 years) (P<0.05). This pattern may reflect reduced viral transmission chains in low-density rural populations, though the specific protective mechanisms require future studies integrating vaccination coverage and population density. Demographically, kindergarten children showed the highest proportion of BV cases and shortest median interval (3.79 years), likely attributable to their initial exposure to group settings, poor hand hygiene compliance, immature immune systems, and heightened oro-manual exploratory behaviors, which collectively increase viral susceptibility (31). In contrast, middle and high school students demonstrated prolonged breakthrough intervals (10.58 and 9.87 years), suggesting age-related enhancements in immune responses or behavioral modifications reducing exposure risks.

Notably, 82.99% of BV cases occurred in single-dose recipients, reaffirming the suboptimal protection conferred by single-dose VarV regimens (32). Vaccination with two doses of VarV can better prevent the occurrence of breakthrough cases of varicella, which is consistent with the results of other studies (23, 33, 34). The mean breakthrough interval for single-dose BV cases (7.50 ± 3.60 years) exceeded reports from Hefei City (6.14 ± 3.14 years) (35) and Beibei District (3.75 ± 1.37 years) (36), potentially explained by immunization age differences. Linear regression analysis of single-dose BV cases revealed a negative correlation between breakthrough intervals and primary immunization age (*r = -0.384, P < 0.05*), indicating that earlier vaccination may prolong the protection duration. However, overly early immunization requires balancing the maturity of the infant immune system against vaccine immunogenicity. While our findings suggest potential advantages of early vaccination, they remain insufficient to challenge the current recommended first-dose window of 12–24 months outlined in the *Chongqing Varicella Vaccination Guidelines* (37). We propose maintaining timely first-dose administration at ≥12 months of age under existing guidelines to establish primary immunization barriers, while advocating for long-term follow-up studies to evaluate the impact of early vaccination on immune memory formation and protection duration.

The study revealed a continuous increase in the proportion of two-dose breakthrough cases, rising from 7.05% in 2019 to 31.28% in 2023, a phenomenon potentially linked to waning immunity or heightened exposure risks in older children (38, 39). Notably, the median breakthrough interval for two-dose cases (3.07 years) was significantly shorter than that for single-dose cases (7.92 years), suggesting that while the two-dose regimen reduces early breakthrough risks, its long-term protective durability requires further evaluation through dynamic antibody monitoring. Further analysis demonstrated a negative correlation between breakthrough intervals and vaccination intervals in two-dose cases (*r = -0.228, P < 0.05*), indicating that longer vaccination intervals corresponded to shorter breakthrough intervals. We hypothesize that extended vaccination intervals may result from incomplete immune memory formation or failure to achieve long-term protective antibody thresholds. Studies suggest that shorter intervals reduce unprotected periods for primary vaccine failures, thereby lowering breakthrough risks (39). These findings highlight the need for varicella booster immunization but emphasize that determining the optimal interval between first and second doses remains a complex immunological challenge, necessitating long-term follow-up to assess dynamic impacts on breakthrough risks.

The findings of this study should be interpreted with caution due to several limitations. First, the absence of systematic control for potential confounders, such as individual immune status, nutritional levels, viral genetic variations, and coinfections, restricts a thorough investigation of BV case mechanisms. Second, owing to challenges in obtaining vaccination rates and town-specific student statistics, we could not evaluate the vaccine’s effectiveness against breakthrough infections or examine regional variations in vaccination patterns. Lastly, COVID-19 pandemic control measures implemented between 2020 and 2022, including school closures and delayed healthcare-seeking behaviors, may have indirectly altered varicella transmission dynamics and breakthrough interval distributions by reducing exposure opportunities or disrupting vaccination services. However, this study did not quantify the specific impact of these pandemic-related confounding factors. Future multi-center prospective studies incorporating virological, immunological, and socio-behavioral data are needed to strengthen the validity of the conclusions.

## 5 Conclusion

In conclusion, the proportion of BV cases among students in Jiulongpo District, Chongqing, has shown a persistent upward trend over time, with two-dose vaccination demonstrating higher effectiveness compared to single-dose regimens. To reduce the risk of breakthrough infections in student populations, we recommend timely administration of the first varicella vaccine dose at 12 months of age, followed by a second booster dose as early as possible.

## Data Availability

The original contributions presented in the study are included in the article/Supplementary Material. Further inquiries can be directed to the corresponding authors.
